# Effects of the Time of Hospice and Palliative Care Enrollment before Death on Morphine, Length of Stay, and Healthcare Expense in Patients with Cancer in Taiwan

**DOI:** 10.3390/healthcare11212867

**Published:** 2023-10-31

**Authors:** Yi-Shu Liao, Wen-Chen Tsai, Li-Ting Chiu, Pei-Tseng Kung

**Affiliations:** 1 Department of Pathology, Taichung Armed Forces General Hospital, National Defense Medical Center, Taichung 411228, Taiwan; yi-shu@803.org.tw; 2 Department of Health Services Administration, College of Public Health, China Medical University, Taichung 406040, Taiwan; wtsai@mail.cmu.edu.tw (W.-C.T.); u9775851@cmu.edu.tw (L.-T.C.); 3 Department of Healthcare Administration, Asia University, No. 500 Lioufeng Road, Wufeng, Taichung 413305, Taiwan; 4 Department of Medical Research, China Medical University Hospital, Taichung 404327, Taiwan

**Keywords:** patient with terminal cancer, hospice care, palliative care, healthcare utilization, morphine dose, total length of hospital stay, total medical expenses

## Abstract

We aimed to investigate the effects of the time from hospice and palliative care enrollment to death on the quality of care and the effectiveness and trend of healthcare utilization in patients with terminal cancer. Data on the cancer-related mortality rates between 2005 and 2018 reported in the National Health Insurance Research Database in Taiwan were obtained. The effect of hospice and palliative care enrollment at different timepoints before death on healthcare utilization was explored. This retrospective cohort study included 605,126 patients diagnosed with terminal cancer between 2005 and 2018; the percentage of patients receiving hospice and palliative care before death increased annually. Terminal cancer patients who enrolled in hospice and palliative care at different timepoints before death received higher total morphine doses; the difference in the total morphine doses between the two groups decreased as the time to death shortened. The difference in the total morphine doses between the groups gradually decreased from 2005 to 2018. The enrolled patients had longer hospital stays; the length of hospital stays for both groups increased as the time to death lengthened, but the difference was not significant. The enrolled patients incurred lower total medical expenses, but the difference between the two groups increased as the time to death shortened.

## 1. Introduction

Cancer remains a major global health challenge, and its high incidence and mortality rates place a huge burden on the society and economy. According to statistics, 24.5 million people worldwide are diagnosed with cancer, and cancer is the leading cause of death in Taiwan with a mortality rate of 220.1 per 100,000 people [1]. The high incidence and mortality rates of cancer have been associated with the highest percentage of medical expenses in Taiwan [2]. Notably, the average medical expenses for cancer patients in Taiwan are projected to increase by 16.4% between 2017 and 2022 [3]. The increasing trend in the expenses suggests that cancer places a gradually increasing burden on the healthcare system.

Previous studies have reported a significant reduction in healthcare resource utilization in patients with terminal cancer who received hospice and palliative care [4]. Hospice and palliative care can significantly reduce the medical expenses. Lowery et al.’s study revealed that patients with ovarian cancer who received hospice and palliative care experienced an average cost reduction of $1285 for expenses related to chemotherapy, hospitalization, and emergency room visits [5]. The total hospitalization expenses decrease significantly in patients with terminal cancer receiving hospice and palliative care within 2 days of hospitalization, and the decrease in the expenses is more pronounced as the number of comorbidities increases [6]. The medical expenses also increase as the cancer progresses [7]. The medical expenses incurred by patients receiving hospice and palliative care are approximately 16.3% lower than those incurred by patients who do not receive such care; the medical expenses incurred by patients receiving hospice and palliative care for more than 30 days are also lower than those for patients receiving the care for less than 30 days [8].

In recent years, Taiwanese people have placed increasing focus on improving their quality of life, and the acceptance of hospice and palliative care is increasing annually, particularly in patients with terminal cancer, whose cancer has either metastasized to distant organs or recurred, making it incurable. Since 1996, Taiwan has been providing home-based hospice care for patients with terminal cancer, and this service was officially included in the National Health Insurance on 1 September 2009 [9]. Taiwan offers three main types of hospice and palliative care: hospice ward, home-based hospice care, and hospice shared care; all of them are covered by the National Health Insurance [10]. Patients with terminal cancer who do not receive hospice and palliative care may incur increased medical expenses due to hospitalization, admission to the intensive care unit (ICU), emergency room visits, and chemotherapy [11].

Research has underscored a U-shaped pattern in the medical resource cost curve for cancer patients. Typically, this cost curve peaks within one year after the initial cancer diagnosis and again within one year before the patient’s demise [12,13]. Furthermore, studies comparing the medical utilization of hospice and palliative care in early versus late stages have found significantly reduced hospitalization costs in the last six months of life for patients who enrolled in hospice and palliative care at earlier stages [14]. Hospice and palliative care can reduce patients’ healthcare utilization and medical expenses before death [15]. The American Society of Clinical Oncology suggests that hospice and palliative care should be provided to patients with terminal cancer as soon as treatment is started, ideally within eight weeks after the diagnosis of terminal cancer [15]. Another study suggested that patients with terminal cancer should start receiving hospice and palliative care 3 months before death; however, patients with terminal cancer usually receive hospice and palliative care one month before death [16]. Compared to non-enrolled patients, enrolled patients have higher healthcare utilization and hospitalization rates within 30 days prior to enrollment but have lower healthcare utilization and hospitalization rates during the interval between enrollment and death [4].

A study conducted in the United States highlighted variations in hospice hospitalization durations. It revealed that for-profit hospice and palliative care institutions tended to have a higher proportion of patients with extended hospitalization periods compared to their non-profit counterparts. This longer hospice and palliative care stay was associated with higher overall costs [17,18]. Furthermore, in Taiwan, it has been demonstrated that patients enrolled in hospice and palliative care incur significantly lower average medical expenses compared to those incurred by non-enrolled patients [11]. The average time from the beginning of hospice and palliative care to death is 26.15 days for patients with cancer who receive inpatient hospice and palliative care and 48.28 days for patients with cancer who receive home-based hospice care [19].

Hospice and palliative care aim to provide physical comfort to patients with terminal cancer and minimize their symptoms of discomfort while considering death as a normal process without hastening or delaying it [10]. Pain relief is one of the most common types of medical treatment for patients with terminal cancer [20], and morphine is the preferred pain medication for those with moderate to severe pain [21]. Morphine, which is an opioid drug commonly used in hospice and palliative care for relieving severe pain and managing symptoms, is widely used as an analgesic for the treatment of severe cancer pain [20]. In hospice and palliative care, the judicious use of opioids is a cornerstone of ethical care for patients with pain stemming from cancer-related symptoms [22]. Specifically, in hospice and palliative care settings, opioids play a central role in addressing pain and dyspnea associated with advanced diseases or the end-of-life process [23]. It is worth noting that recent shifts in medical policies related to opioid prescription, coupled with increased scrutiny of healthcare providers, may inadvertently hinder patient access to these vital medications. Consequently, this can result in untreated symptoms and increased distress, intensifying patients’ end-of-life suffering [24,25]. Notably, some caregivers have expressed apprehension about opioid use, stemming from an incomplete understanding of opioids and concerns regarding potential patient addiction [26]. In terms of pain management, hospice and palliative approaches are becoming increasingly critical when providing care to patients with terminal cancer. Hospice and palliative care not only provide physical and mental comfort but also reduce medical expenses and unnecessary treatment [10]. Taiwan’s healthcare system is actively committed to facilitating hospice and palliative care for patients with terminal cancer and provides relevant financial support to them [9].

Cancer not only causes a serious impact on an individual’s life but also places a huge burden on society and the healthcare system. Given these circumstances, hospice and palliative care, which provide physical comfort and pain relief while considering death as a natural process, have become important options for patients with terminal cancer. No study has investigated the appropriate timing for providing hospice interventions and analyzed whether patients with terminal cancer should enroll in hospice and palliative care before death, including their effects on the length of hospital stay, morphine dose, healthcare utilization, and medical expenses. This study aimed to investigate the effects of enrolling in hospice and palliative care at different timepoints before death on the healthcare utilization rates of patients with terminal cancer.

## 2. Materials and Methods

### 2.1. Study Population

**Figure 1:**
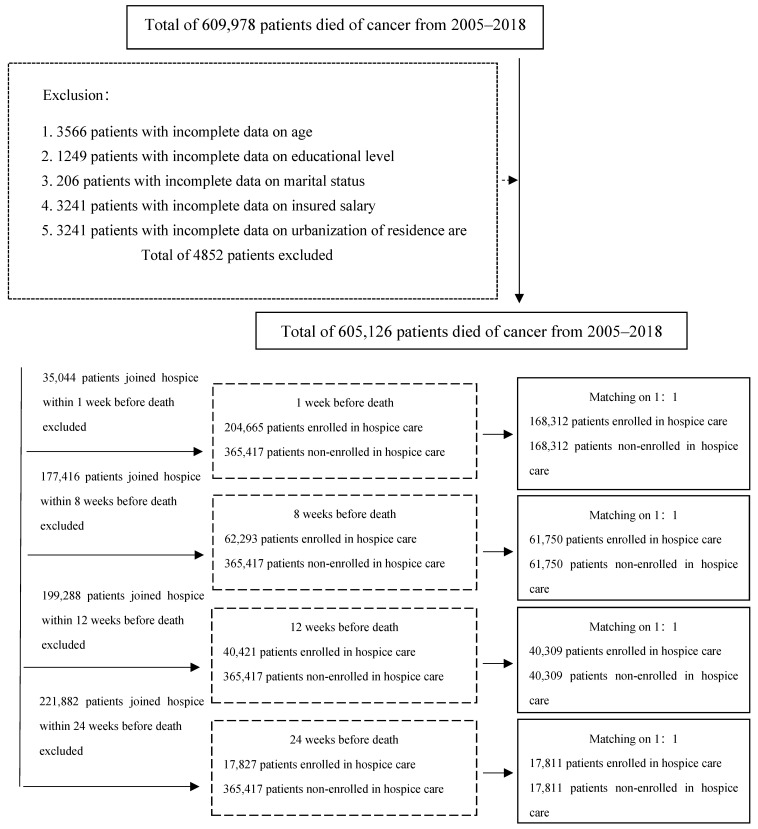
Flowchart of research subject selection.

This study included Taiwanese patients whose primary or secondary cause of death documented from 2005 to 2018 was cancer. The International Classification of Diseases, 9th Revision, Clinical Modification (ICD-9-CM) codes 140–208 and ICD-10-CM codes C00–C97 were used to classify patients with cancer. All patients, excluding those with missing values on the study variables, were selected; a total of 605,126 patients died of cancer. To minimize the selection bias between the patients with terminal cancer who received hospice and palliative care and those who did not, a year-by-year propensity score matching on a 1:1 ratio was conducted between enrolled and non-enrolled patients according to gender, age, severity of comorbidities, and types of cancer. Figure 1 shows the data extraction process.

The personal data of all participants were de-identified to maintain confidentiality; the study was approved by the Research Ethics Committee of Jen-Ai Hospital (institutional review board No.: 110-38).

### 2.2. Sources of Data

In this retrospective cohort study, the secondary data from the National Health Insurance Research Database (NHIRD) of the Ministry of Health and Welfare of Taiwan were analyzed. Data on the medical staff were obtained from the registry for medical personnel (PER) and the cancer registration database of the Health Promotion Administration between 2005 and 2018. Data on the cause of death and household registration status were obtained from the Ministry of the Interior, while the educational level and marital status were extracted from the household registration data.

The National Health Insurance has been implemented since March 1995; it covered 99.93% of Taiwanese citizens at the end of 2022 [27]. The National Health Insurance, which is a mandatory national insurance system, provides comprehensive medical coverage for all citizens and legal residents. The system covers a wide range of medical expenses, including drug prescriptions, treatment, surgeries, outpatient visits, hospitalization, and emergency examinations and treatment, as well as treatment for patients with cancer. The health insurance-related data provided by the National Health Insurance have become an important source of empirical data for healthcare research and are often used as a reference for the formulation of healthcare policies.

### 2.3. Study Design

This study compared patients with terminal cancer who enrolled in hospice and palliative care with non-enrolled patients to examine the effects of different lengths of hospice and palliative care on the changes in healthcare utilization in patients with terminal cancer, including the total morphine dose used for pain management, total length of hospital stay, and total medical expenses before death.

### 2.4. Descriptions of Variables

The effects of the length of hospice and palliative care on the morphine dose in patients with terminal cancer were explored. The dependent variable was the morphine dose, the independent variables were the enrollment in hospice and palliative care and the length of care, and the control variables were the patient’s baseline characteristics (gender, age, and marital status), economic factor (monthly income), environmental factor (degree of urbanization of the place of residence), health status (severity of comorbidities), type of cancer, enrollment in multidisciplinary cancer care, characteristics of medical institutional characteristics (level of medical institution and ownership of the institution), characteristics of the attending physician (gender and age), and year of patient’s death.

When investigating the effects of the length of hospice and palliative care on the total length of hospital stay in patients with terminal cancer, the dependent variable was the total length of hospital stay, the independent variables were the enrollment in hospice and palliative care and the length of care, and the control variables were the same as above.

When examining the effects of the length of hospice and palliative care on the total medical expenses incurred by patients with terminal cancer, the dependent variable was the total medical expenses, the independent variables were the enrollment in hospice and palliative care and the length of care, and the control variables were the same as above.

The length of hospice and palliative care included that of the hospital- and home-based care provided to patients with terminal cancer who enrolled in the care hospice and palliative program. The patients were determined to enroll in hospice and palliative care if the standard reporting code for a hospice-related payment was captured (Supplementary Table S1). This study aimed to explore the time efficiency of hospice and palliative care for patients with terminal cancer. Therefore, this study observed whether the study patients enrolled in hospice and palliative care at 1, 4, 8, 12, and 24 weeks before death.

The dependent variables included total morphine dose, total length of hospital stay, and total medical expenses. The individual total morphine dose was calculated for each observation period. A medication order for morphine is shown in Supplementary Table S2. The individual total length of hospital stay was also calculated for each observation period. Moreover, the individual total medical expenses, including co-payments, for outpatient visits, emergency room visits, and hospitalization were estimated for each observation period.

The control variables included the characteristics of the patient, economic factors, environmental factors, health status, type of cancer, multidisciplinary cancer care, characteristics of the attending physician, characteristics of the primary healthcare provider (level of and ownership of the institution), and year of patient’s death. The characteristics of a patient included gender, age, and marital status. Marital status was categorized into single, married, divorced, or widowed, according to the marital status in the household registration data.

The financial status of a patient was determined based on the monthly salary, which was divided into ≤NTD17,280; NTD17,281–22,800; NTD22,801–28,800; NTD28,801–36,300; NTD36,301–45,800; NTD45,801–57,800; and ≧NTD57,800 (1 US dollar = 30 NTD). The patient’s residential environment was classified according to the degree of urbanization based on the criteria established by Liu et al., with the first level being the highest degree of urbanization and the seventh level being the lowest degree of urbanization [28].

The health status of a patient was determined based on the Charlson Comorbidity Index (CCI) modified by Deyo et al., which classifies comorbidities into 17 distinct categories [29]. The codes for the patient’s primary and secondary diagnoses (ICD-9-CM) (specifically for those with more than two outpatient visits or one hospitalization) were converted to numerical weighted scores, and the weighted scores were summed to calculate Deyo’s CCI score. The cancer-related diagnoses were not included in the calculation of the CCI score to avoid double counting. The medical diagnostic data of each patient within 2 years before death were converted to the CCI, and a higher CCI score indicated a more severe comorbidity. We ascertained whether a study patient was alive or dead and the date of death from the data obtained from the Ministry of the Interior.

Based on the cancer mortality data obtained from the Health Promotion Administration’s cancer registration database, this study categorized the primary causes of death into lung cancer, liver cancer, colorectal cancer, female breast cancer, oral cancer, prostate cancer, gastric cancer, pancreatic cancer, esophageal cancer, cervical cancer, other types of cancer, and multiple cancers.

This study identified whether any of the patients enrolled in multidisciplinary cancer care by reviewing patient reports of fees related to cancer treatment planning and consultation (47079B) prior to their enrollment in hospice and palliative care (for the enrolled patients) or death (for non-enrolled patients).

The attending physician of a patient was defined as the physician who reviewed the patient’s medical records within 3 months prior to the enrollment in hospice and palliative care (for the enrolled patient) or death (for the non-enrolled patient) and provided the highest quantity of outpatient and inpatient treatments. Meanwhile, the primary medical institution for a patient with terminal cancer who enrolled in hospice and palliative care was determined as the facility where the patient initially enrolled in the care program. By contrast, the primary medical institution for a patient with terminal cancer who did not enroll in hospice and palliative care was identified as the facility with the highest numbers of outpatient visits and hospitalization within 3 months prior to death. The primary medical institutions were divided into four levels: medical centers, regional hospitals, district hospitals, and clinics. The primary medical institutions were divided into two categories based on institutional ownership: public hospitals and private hospitals.

### 2.5. Statistical Analysis

Descriptive statistics, including the number and percentage of cancer patients, were used to present the distribution of participants for each independent variable (such as enrollment in hospice and palliative care and length of care), under different control variables (such as the characteristics of patients, economic factors, environmental factors, severity of comorbidities, type of cancer, enrollment in a multidisciplinary team, characteristics of the attending physician, characteristics of the primary medical institutions, and year of patient’s death).

This study used a year-by-year propensity score matching on a 1:1 ratio to reduce the selection bias between the enrolled and non-enrolled patients. For the logistic regression model, enrollment in hospice and palliative care was set as the dependent variable, whereas gender, age, severity of comorbidities, and type of cancer were set as independent variables, followed by year-by-year propensity score matching.

As the total morphine dose, total length of hospital stay, and total medical expenses may be non-normally distributed, the Mann–Whitney test and Kruskal–Wallis test were used to determine whether the period of enrollment in hospice and palliative care differed according to the total morphine dose, total length of hospital stay, and total medical expenses incurred at 1, 4, 8, 12, and 24 weeks before death under the different control variables described above. The total morphine dose, total length of hospital stay, and total medical expenses incurred at 1, 4, 8, 12, and 24 weeks before death were set as dependent variables after converting their values into a natural logarithmic form. The enrollment in hospice and palliative care incurred at 1, 4, 8, 12, and 24 weeks before death were retained as the independent variables. After controlling for the control variables mentioned above, the effects of the length of hospice and palliative care on the total morphine dose, total length of hospital stay, and total medical expenses incurred by patients with terminal cancer were investigated by performing a multiple regression analysis using the generalized estimating equation on the matched participants. The results of the statistical analysis were expressed as ratios along with the corresponding 95% confidence intervals (CIs).

The SAS statistical software, version 9.4 (SAS Institute Inc., Cary, NC, USA), was used to perform the descriptive and inferential statistical analyses. All tests were two-tailed, and a *p*-value of <0.05 was considered significant.

## 3. Results

### 3.1. Trend of Hospice and Palliative Care Enrollment before Death in Patients with Terminal Cancer

In total, 609,978 patients died of cancer from 2005 to 2018, while 4852 patients with incomplete data on monthly salary, educational level, and medical institution were excluded from this study. Hence, only 605,126 patients were included in the final analysis. Supplementary Table S3 shows that the percentage of patients with terminal cancer receiving hospice and palliative care before death increased annually, with 17.79–22.5% in 2005–2010, followed by significant annual increases after 2010 to 62.51% in 2018, showing a trend of substantial increase.

In Supplementary Table S4, a comprehensive overview is presented. Prior to matching in our study, 39.61% of terminal cancer patients in this study had enrolled in hospice and palliative care before their demise. Notably, the data reveal that a higher proportion of women, constituting 43.52%, had accessed hospice and palliative care compared to men, whose participation rate was 37.31%. Regarding age groups, those aged 55–64 exhibited the highest participation rate at 43.40%, while the participation rate among individuals aged 75–85 was the lowest, standing at 36.87%. Educational levels had a discernible influence, with higher education correlating with a higher rate of hospice and palliative care enrollment, particularly those with a college education or above, accounting for the highest rate at 44.57%. Marital status also demonstrated variability in hospice and palliative care participation, with married individuals exhibiting the lowest enrollment rate at 39.10%, whereas unmarried individuals recorded the highest enrollment rate at 41.86%. In addition, 49.90% of patients diagnosed with pancreatic cancer engaged in hospice and palliative care, whereas those with prostate cancer displayed the lowest participation rate at 34.37%. The involvement of patients in a multi-specialty cancer care team yielded a considerably higher hospice and palliative care enrollment rate, reaching 52.85%, compared to those who had not engaged with such teams, with a rate of 35.76%. Moreover, patients whose principal healthcare institution was a medical center exhibited the highest proportion, with an enrollment rate of 41.33%.

To reduce the selection bias between the enrolled and non-enrolled patients, patients who enrolled in hospice and palliative care for less than 1, 4, 8, 12, and 24 weeks, respectively, before conducting the year-by-year propensity score matching on a 1:1 ratio were excluded (Supplementary Table S5). Eventually, two groups of patients without significant differences in gender, age, severity of comorbidities, and type of cancer were obtained. Subsequently, the differences in the total morphine dose, total length of hospital stay, and total medical expenses incurred by the two groups were compared.

### 3.2. Comparison of the Differences in the Total Morphine Dose, Total Length of Hospital Stay, and Total Medical Expenses Incurred by Patients with Terminal Cancer Who Enrolled and Those Who Did Not Enroll in Hospice and Palliative Care at 1, 4, 8, 12, and 24 Weeks before Death

As shown in Table 1, the total morphine dose, total length of hospital stay, and total medical expenses decreased as the time to death shortened in both enrolled and non-enrolled patients. For example, from 1 week to 24 weeks before death, the morphine dose increased from an average of 175.27 ± 446.98 mg to an average of 4048.78 ± 11,648.15 mg (*p* < 0.05) in the enrolled patients and increased from an average of 65.08 ± 254.39 mg to an average of 758.47 ± 3941.51 mg (*p* < 0.05) in non-enrolled patients. Then, the total length of hospital stay increased from an average of 5.46 ± 2.58 days to an average of 50.66 ± 46.09 days (*p* < 0.05) in enrolled patients and increased from an average of 4.39 ± 2.99 days to an average of 32.87 ± 32.94 days (*p* < 0.05) in non-enrolled patients. Lastly, the total medical expenses increased from an average of NT$26,683 ± 28,517 to an average of NT$365,251± 314,337 (*p* < 0.05) in enrolled patients and increased from an average of NT$43,714 ± 55,888 to NT$389,052 ± 405,138 (*p* < 0.05) in non-enrolled patients.

### 3.3. Effects of Hospice and Palliative Care Enrollment before Death on the Total Morphine Dose, Total Length of Hospital Stay, and Total Medical Expenses Incurred by Patients with Terminal Cancer

After controlling for other relevant variables, the multiple regression model showed that the difference in the total morphine dose between the enrolled and non-enrolled patients increased as the length of hospice and palliative care increased; however, the difference in the total length of hospital stay gradually decreased, while the difference in the total medical expenses remained a non-significant change (Table 2). The total morphine dose for the patients with terminal cancer who enrolled in hospice and palliative care 1 week before death was 19.29 times higher than that for the non-enrolled patients (95% CI: 18.74–19.84). As the length of hospice and palliative care increased, the total morphine dose of the patients who enrolled in hospice and palliative care 24 weeks before death was 23.49 times higher than that of the non-enrolled patients (95% CI: 21.20–26.03). The total length of hospital stay of the patients who enrolled in hospice and palliative care 1 week before death was 1.93 times higher than that of the non-enrolled patients (95% CI: 1.90–1.97). As the length of hospice and palliative care increased, the ratio of the total length of hospital stay between the two groups gradually decreased, with the total length of hospital stay of the patients who enrolled in hospice and palliative care 24 weeks before death being 1.57 times higher than that of the non-enrolled patients (95% CI: 1.50–1.65). Meanwhile, the total medical expenses incurred by patients who enrolled in hospice and palliative care 1 week before death were 0.79 times higher than those of non-enrolled patients (95% CI: 0.78–0.81); the difference in the total medical expenses incurred by the two groups slightly increased as the length of hospice and palliative care increased, with the total medical expenses incurred by patients who enrolled in hospice and palliative care 24 weeks before death being 0.73 times higher than those of non-enrolled patients (95% CI: 0.70–0.76).

### 3.4. Year-to-Year Trends of Total Morphine Dose, Total Length of Hospital Stay, and Total Medical Expenses

**Figure 2:**
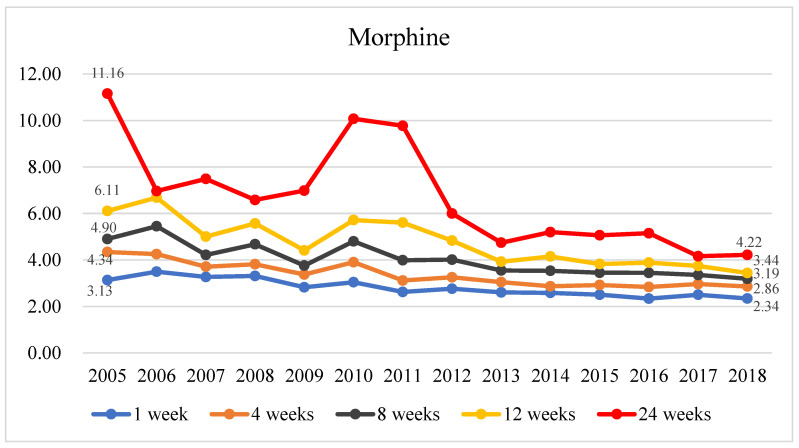
The historical trend of the ratio of mean morphine consumption between terminal cancer patients enrolled in hospice care and those not enrolled, at 1, 4, 8, 12, and 24 weeks before death.

As shown in Figure 2 and Supplementary Table S6, the enrolled patients received higher total morphine doses than those received by the non-enrolled patients. The ratio of the average total morphine dose of patients who enrolled in hospice and palliative care at 1, 4, 8, 12, and 24 weeks before death to that of non-enrolled patients decreased as the year of death approached (from 2005 to 2018); this finding indicates that the ratio of the total morphine dose between the two groups decreased as the year of death approached. Regardless of the length of hospice and palliative care, the average total morphine dose reduced from 2005 to 2018, with the enrolled patients experiencing significantly greater reductions than those experienced by the non-enrolled patients. The total morphine doses decreased from an average of 243.93 mg in 2005 to an average of 128.20 mg in 2018 in patients who enrolled 1 week before death and decreased from an average of 77.82 mg in 2005 to an average of 54.75 mg in 2018 in non-enrolled patients, with the ratio decreasing from 3.13 in 2005 to 2.34 in 2018. The total morphine doses dropped from an average of 5727.91 mg in 2005 to 2644.47 mg in 2018 in patients who enrolled 24 weeks before death and increased from an average of 513.42 mg in 2005 to an average of 626.86 mg in 2018 in non-enrolled patients, with the ratio decreasing from 11.16 in 2005 to 4.22 in 2018.

**Figure 3:**
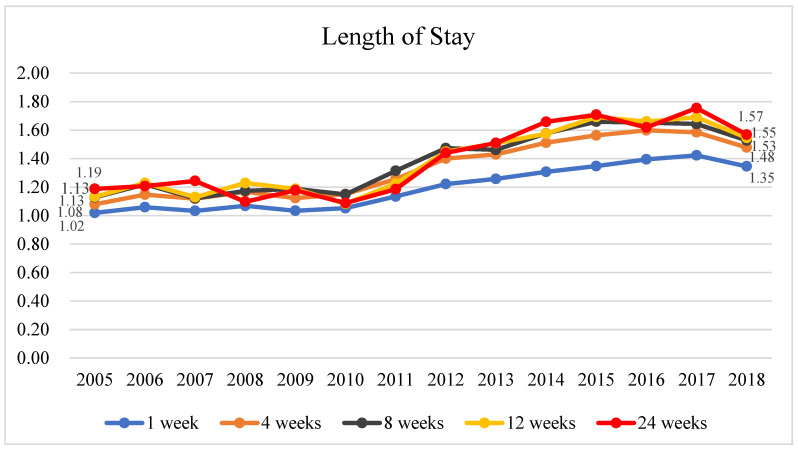
The historical trend of the ratio of the mean total length of stay between terminal cancer patients enrolled in hospice care and those not enrolled, at 1, 4, 8, 12, and 24 weeks before death.

Figure 3 and Supplementary Table S7 show that the enrolled patients had a longer hospital stay than the non-enrolled patients. Regardless of the length of hospice and palliative care, the ratio of the total length of hospital stay of patients who enrolled at 1, 4, 8, 12, and 24 weeks before death to that of non-enrolled patients slightly increased as the year of death approached, with slightly different year-to-year trends for different timepoints before death. For instance, the ratio of the total length of hospital stay of the patients who enrolled 1 week before death to that of the non-enrolled patients remained roughly similar between 2005 and 2010, with the values ranging from 1.02 to 1.07, while the ratio steadily increased to 1.35 from 2010 to 2018. The trends at 4, 8, 12, and 24 weeks before death were slightly different but generally similar to the trend at 1 week before death. From 2005 to 2018, the lengths of hospital stay slightly increased from an average of 5.00 days to an average of 5.37 days in patients who enrolled 1 week before death but decreased from an average of 4.90 days to an average of 3.99 days in non-enrolled patients. The lengths of hospital stay slightly decreased from an average of 49.05 days to an average of 48.46 days in patients who enrolled at 24 weeks before death and decreased from an average of 41.30 days to an average of 30.90 days in non-enrolled patients. The average total length of hospital stay slightly decreased as the year of death approached in non-enrolled patients.

**Figure 4:**
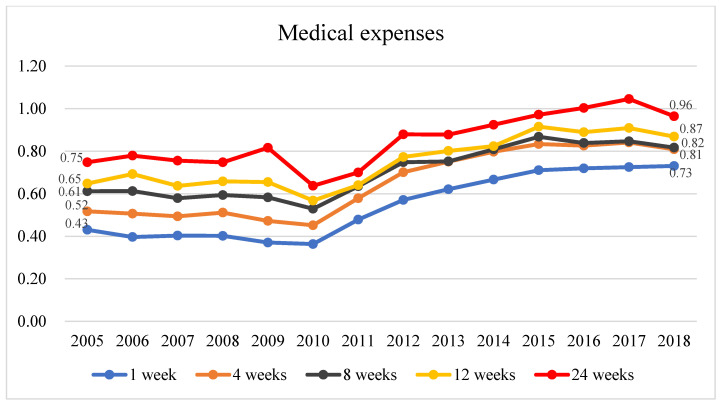
The historical trend of the ratio of the mean total medical expenses between terminal cancer patients enrolled in hospice care and those not enrolled, at 1, 4, 8, 12, and 24 weeks before death.

Figure 4 and Supplementary Table S8 show that the enrolled patients incurred lower total medical expenses than those incurred by the non-enrolled patients. Regardless of the length of hospice and palliative care, as the year of death approached, the ratio of the total medical expenses incurred by the patients who enrolled in hospice and palliative care at 1, 4, 8, 12, and 24 weeks before death to those of the non-enrolled patients gradually decreased from 2005 to 2018, with similar year-to-year trends for the total medical expenses incurred by patients with terminal cancer at different timepoints before death. For example, the ratio of the total medical expenses incurred by the patients who enrolled 1 week before death to those of the non-enrolled patients remained roughly similar between 2005 and 2010 with the values ranging from 0.36 to 0.43, but the ratio increased to 0.73 from 2010 to 2018. The trends at 4, 8, 12, and 24 weeks before death were similar to the trend at 1 week before death. This finding indicates that the difference in the total medical expenses incurred by the two groups slightly decreased as the year of death approached. Meanwhile, an increasing trend was observed in the average total medical expenses incurred by the enrolled and non-enrolled patients. Nevertheless, the average total medical expenses increased by a greater magnitude in enrolled patients than they did in non-enrolled patients; the total medical expenses increased from an average of NT$17,189 to an average of NT$33,659 in patients who enrolled at 1 week before death and from an average of NT$39,925 to an average of NT$46,062 in non-enrolled patients; meanwhile, the total medical expenses increased from an average of NT$275,196 to an average of NT$407,083 in patients who enrolled at 24 weeks before death and increased from an average of NT$ 367,907 to an average of NT$422,189 in non-enrolled patients.

## 4. Discussion

The percentage of patients with terminal cancer receiving hospice and palliative care before death increased annually from 17.79% in 2005 to 62.51% in 2018, with particularly significant increases after 2010 due to the inclusion of hospice and palliative care in the National Health Insurance of Taiwan since 2009 [9]. Therefore, hospice and palliative care are becoming more accessible to patients with terminal cancer in Taiwan, allowing hospice and palliative care teams to intervene in cancer-related treatment promptly. A study in the United States (US) indicated that the costs of cancer care are divided into an initial phase, a continuing phase, and a terminal phase; the terminal phase includes the provision of intensive care and hospice and palliative care within 12 months before death [30]. This study focused on the hospice and palliative care provided at different timepoints within 6 months before death for further analysis.

The patients with terminal cancer who enrolled in hospice and palliative care at different timepoints before death received higher total morphine doses than those received by the non-enrolled patients, thus suggesting that hospice and palliative care teams focus on managing patients’ pain and thus increase the morphine doses. At different timepoints before death in the same year, the difference in the total morphine dose between the enrolled and non-enrolled patients decreased as the time to death shortened. Therefore, delayed enrollment in hospice and palliative care results in reduced effectiveness in the total morphine dose. From 2005 to 2018, the difference in total morphine dose between the enrolled and non-enrolled patients gradually reduced, with the greatest reduction at 24 weeks before death (from 11.16 in 2005 to 4.22 in 2018). Some studies have shown an increase in the usage rate of analgesics and opioids among patients with terminal cancer following their enrollment in hospice and palliative care [31,32]. The goal of morphine use is to minimize discomfort in patients with terminal cancer [10,33]. In other words, enrolling in hospice and palliative care at an earlier stage enables the healthcare team to promptly evaluate and customize the morphine dosage based on the patient’s condition to ensure effective pain management while preventing the misuse of morphine, ultimately enhancing the quality of care [10,20]. With the promotion and implementation of hospice and palliative care, professional medical teams can enhance the quality of life of patients with terminal cancer. This goal was achieved by prioritizing the management of cancer-related pain and optimizing the utilization of morphine, all while ensuring the patient’s needs are met without any misuse of pain medications. As a result, the total morphine dose used by patients with terminal cancer who enrolled in hospice and palliative care decreased in recent years, thus suggesting an increase in quality of care.

In the same year of death, the patients with terminal cancer who enrolled in hospice and palliative care at different timepoints before death had a longer hospital stay than that of the non-enrolled patients. At different timepoints before death in the same year, the total length of hospital stay increased as the time of death approached in both enrolled and non-enrolled patients, but the difference between the two groups was not significant, which is inconsistent with the findings of previous studies. For example, studies from the US noted that patients who received hospice and palliative care consultations had shorter hospital stays within 3 months before death than patients who did not [34,35]. Moreover, other studies from the US showed that the longer the length of hospice and palliative care, the greater the reduction in the length of hospital stay [36]. Both findings are contrary to our results. The differences may be attributed to the fact that patients in the US are transferred to outpatient hospice and palliative care clinics more often than to inpatient hospice wards after enrollment in hospice and palliative care [37]. Furthermore, most patients in the US receive hospice and palliative care in their homes, to minimize the medical expenses, travel distance, and time cost [37]. By contrast, most patients with terminal cancer in Taiwan receive inpatient hospice and palliative care [11], suggesting that Taiwan can enhance the promotion of outpatient hospice and palliative care clinics or non-hospital-based hospice care (home-based hospice care or institutional hospice care) to reduce the use of inpatient hospice and palliative care. In this study, the total length of hospital stay included the length of stay in both acute wards and hospice wards for the enrolled patients but only included the length of stay in acute wards for non-enrolled patients, resulting in a longer hospital stay in enrolled patients than in non-enrolled patients. The number of hospice wards in Taiwan remains insufficient; thus, some patients who enrolled in hospice and palliative care are admitted to the acute wards. However, the data from the NHIRD could not distinguish whether the enrolled patients stayed in an acute ward or a hospice ward when they were hospitalized, and this is one of the limitations of this study.

On the contrary, Taiwan has implemented a diagnosis-related group (DRG) payment system for some diseases since 2010, which is a system that provides hospitals with a fixed payment for some inpatients [38]. Therefore, hospitals reduce the DRG patients’ length of hospital stay to lower medical expenses [39,40]. Hospitals in Taiwan have probably intensified their oversight of acute wards due to the implementation of the DRG payment system. Consequently, the length of hospital stay for non-enrolled patients has exhibited an annual slight decline since 2010. Conversely, the length of hospital stay for enrolled patients has shown a slight annual increase due to the inclusion of hospice and palliative care in the National Health Insurance since 2009.

Concerning the effects of hospice and palliative care enrollment on the total medical expenses, patients with terminal cancer who enrolled in hospice and palliative care at different timepoints before death incurred lower total medical expenses than those incurred by non-enrolled patients, and the results were consistent across years. Studies have concluded that enrollment in hospice and palliative care is significantly associated with a reduction in total medical expenses [6,39,40,41,42], and this is consistent with our findings.

Another study reported that an increase in ICU utilization resulted in an increase in the total expenses and that the expenses of ICU utilization strongly correlated with the total healthcare expenses [43]. These findings are consistent with those of previous studies [32,44]. A Taiwanese study also revealed that the expensive medical expenses incurred by patients with cancer are associated with non-enrollment in hospice and palliative care, the length of hospital stay, and ICU treatment [11]. Moreover, previous studies have noted that patients with terminal cancer who received hospice and palliative care have lower ICU utilization than those utilized by non-enrolled patients [32].

In terms of the total medical expenses, the difference between the expenses incurred by enrolled and non-enrolled patients gradually decreased from 2005 to 2018, but the difference between the two groups increased as the time to death shortened. In 2018, patients who enrolled in hospice and palliative care 1 week before death achieved a remarkable reduction of 27% in the average total medical expenses, while those who enrolled in hospice and palliative care 24 weeks before death only achieved a reduction of 3.5% in the average total medical expenses; this discrepancy highlights the comparatively limited cost-saving effectiveness associated with early enrollment in hospice and palliative care, which is similar to the findings of previous studies [40]. The effect of cost reduction was less pronounced as the length of hospice and palliative care increased, with a savings of 25% in the last month before death and no savings at all in the last 12 months before death [45]. Another Taiwanese study also found that the effect of cost reduction was less pronounced as the length of hospice and palliative care increased [46]. This discovery can be elucidated by the fact that the medical expenses incurred by the non-enrolled patients tend to significantly increase in the terminal phase prior to their death owing to their frequent admissions to the ICU and the substantial emergency treatments necessitated by their deteriorating health condition. Therefore, enrollment in hospice and palliative care one week before death is the most effective approach. In recent years, the perspectives of patients and their family members have undergone a transformation, with a growing willingness to engage in hospice and palliative care at an earlier stage. This proactive approach is geared towards reducing and preventing the social burden placed on caregivers. This shift in patient and family preferences may be a substantial factor contributing to the notable increase in the total medical expenses incurred by enrollees.

Early enrollment in hospice and palliative care facilitates a more deliberate assessment of the patient’s emergency care needs, such as intensive care and emergency-related medical treatment. However, the government should intensify its focus on enhancing the quality of care and healthcare utilization and improving the communication skills of healthcare professionals to ensure that physicians and relevant professionals can engage in early-stage discussions about hospice and palliative care for patients with cancer. The guidance from professional medical teams not only permits a judicious assessment of the necessity for pharmaceutical interventions and prevents medication misuse but also contributes to the alleviation of pain and improvement of the quality of life of terminal cancer patients in their final stages while maximizing the utilization of medical resources. The findings of this study underscore the significance of hospice and palliative care in enhancing the quality of life for terminal cancer patients and fostering improved communication among patients, their families, and the healthcare team. Consequently, we advocate for the government’s continued efforts to expand access to hospice and palliative care for cancer patients in need. While the early initiation of hospice and palliative care may entail increased costs, it emerges as a superior choice for family caregivers, a facet often unseen by clinical medical providers. The appropriate use of morphine and its prudent management necessitate collective deliberation and consideration by both medical professionals and patients. Taiwan’s experience with hospice and palliative care, which has marginally extended the duration of patient hospitalizations, prompts a vital concern of the government—promoting and reinforcing home hospice and palliative care. The ultimate goal is to enable patients to receive more suitable care in the comfort of their homes, thereby diminishing the imperative for hospitalization. Hospice and palliative care effectively reduce the necessity for intensive and costly medical interventions, such as emergency room visits, inpatient hospital stays, and admission to the intensive care unit (ICU). This, in turn, results in diminished healthcare utilization and reduced overall healthcare costs, aligning with healthcare policies and the prudent allocation of resources.

Our study, centered on hospice and palliative care, presents a comprehensive and patient-centric approach to end-of-life care specifically tailored to meet the needs of terminal cancer patients. It encourages patients to harmonize their medical care with their deeply held values. Furthermore, hospice and palliative care profoundly impact healthcare policy and resource allocation by curbing healthcare utilization and expenses. In sum, hospice and palliative care strive to afford terminal cancer patients the highest possible quality of life during the remaining phase of their journey, offering compassionate and dignified support to both patients and their families. Our findings provide an empirical basis for enrolling patients with terminal cancer in hospice and palliative care and can serve as a crucial reference for the formulation of relevant healthcare policies.

## 5. Conclusions

In terms of the total morphine dose, patients with terminal cancer who enrolled in hospice and palliative care at different timepoints before death received higher total morphine doses than those received by non-enrolled patients, indicating the hospice and palliative care teams’ dedicated focus on achieving effective pain management. Moreover, the difference in the total morphine doses between enrolled and non-enrolled patients decreased as the time to death shortened, suggesting that later enrollment in hospice and palliative care is less effective in terms of the total morphine dose. The difference in the total morphine doses between enrolled and non-enrolled groups gradually decreased from 2005 to 2018. Concerning the total length of hospital stay, the patients with terminal cancer who enrolled in hospice and palliative care at different timepoints before death had longer hospital stays than the non-enrolled patients. Both groups showed an increase in the total length of hospital stay as the time to death lengthened, but the magnitude of the difference between the two groups was small; this finding can be attributed to the predominant inpatient nature of hospice and palliative care in Taiwan. The total medical expenses were lower for patients with terminal cancer who enrolled in hospice and palliative care at different timepoints before death than the expenses incurred by non-enrolled patients. Nevertheless, the difference in the total medical expenses incurred by the two groups increased as the time to death shortened, implying that later enrollment of patients with terminal cancer in hospice and palliative care may yield greater cost-effectiveness.

### 5.1. Strengths of the Study

To our knowledge, this study is the first to examine the morphine dose, length of hospital stay, and total medical expenses incurred by patients with terminal cancer and evaluate the trends of morphine dose, length of hospital stay, total medical expenses, and effectiveness of hospice and palliative care. These are the hallmarks of this study. In addition, this study utilizes a database that contains several years of data from the entire Taiwanese population, allowing an extensive understanding of each individual’s medical treatment status and tracking each status for more than 10 years, which makes the findings more representative.

### 5.2. Limitations of the Study

The data used in this study were obtained from a secondary database that contains only the information reported by the healthcare organizations for expense declaration. Therefore, this study does not include the analysis of all relevant factors, such as the patient’s lifestyle and health behaviors. As a result, we were unable to acquire information about the potential confounders, such as the symptoms, family history, and lifestyle factors (including diet and physical activity). In addition, we are unable to investigate the influence of the primary caregiver on the patient’s enrollment in hospice and palliative care and cannot distinguish whether the enrolled patients stayed in the acute wards or the hospice wards. In Taiwan, patients diagnosed with cancer may seek other self-funded treatment, potentially resulting in an underestimation of the actual medical expenses. Lastly, the healthcare system in Taiwan has characteristics distinct from those of other countries, and the provision of hospice and palliative care in Taiwan carries its unique attributes. Therefore, our findings may not apply to all cancer patients worldwide.

## Figures and Tables

**Table 1:** Comparison of the differences in the total morphine dose, total length of hospital stay, and total medical expenses incurred by patients with terminal cancer who enrolled and those who did not enroll in hospice care at 1, 4, 8, 12, and 24 weeks before death.

Variables	1 Week before Death						
	N	%	Mean	SD	Median	IQR	*p*-Value *
Total morphine dose	336,624	100.00	120.18	367.81	8.75	101.11	
Hospice care							<0.001
not enroll	168,312	50.00	65.08	254.39	0.00	24.50	
enroll	168,312	50.00	175.27	446.98	40.00	195.00	
Total length of stay	336,624	100.00	4.92	2.84	7.00	5.00	
Hospice care							<0.001
not enroll	168,312	50.00	4.39	2.99	6.00	6.00	
enroll	168,312	50.00	5.46	2.58	7.00	2.00	
Total medical expenses	336,624	100.00	35,198	45,176	24,766	34,162	
Hospice care							<0.001
not enroll	168,312	50.00	43,714	55,888	31,850	43,083	
enroll	168,312	50.00	26,683	28,517	19,988	25,514	
**Variables**	**4 Weeks before Death**						
	**N**	**%**	**Mean**	**SD**	**Median**	**IQR**	*p*-Value *
Total morphine dose	217,080	100.00	534.47	1556.38	30.00	460.00	
Hospice care							<0.001
not enroll	108,540	50.00	257.71	904.95	0.00	90.00	
enroll	108,540	50.00	811.24	1967.87	170.00	880.92	
Total length of stay	217,080	100.00	16.36	11.41	17.00	25.00	
Hospice care							<0.001
not enroll	108,540	50.00	13.51	10.93	12.00	21.00	
enroll	108,540	50.00	19.21	11.16	23.00	21.00	
Total medical expenses	217,080	100.00	111,069	129,244	77,764	107,712	
Hospice care							<0.001
not enroll	108,540	50.00	127,744	151,960	89,104	127,388	
enroll	108,540	50.00	94,394	98,793	69,355	88,763	
**Variables**	**8 Weeks before Death**						
	**N**	**%**	**Mean**	**SD**	**Median**	**IQR**	*p*-Value *
Total morphine dose	123,500	100.00	1073.91	3250.31	40.00	800.00	
Hospice care							<0.001
not enroll	61,750	50.00	457.65	1805.85	0.00	130.00	
enroll	61,750	50.00	1690.16	4136.25	255.00	1751.67	
Total length of stay	123,500	100.00	25.14	20.07	21.00	34.00	
Hospice care							<0.001
not enroll	61,750	50.00	20.19	17.80	16.00	27.00	
enroll	61,750	50.00	30.08	20.97	28.00	39.00	
Total medical expenses	123,500	100.00	177,603	189,518	130,278	167,785	
Hospice care							<0.001
not enroll	61,750	50.00	199,820	223,050	144,311	195,300	
enroll	61,750	50.00	155,386	145,246	119,315	143,970	
**Variables**	**12 Weeks before Death**						
	**N**	**%**	**Mean**	**SD**	**Median**	**IQR**	*p*-Value *
Total morphine dose	80,618	100.00	1518.72	4806.72	40.00	962.65	
Hospice care							<0.001
not enroll	40,309	50.00	581.74	2465.62	10.00	150.00	
enroll	40,309	50.00	2455.69	6194.71	285.00	2444.09	
Total length of stay	80,618	100.00	30.86	26.72	24.00	38.00	
Hospice care							<0.001
not enroll	40,309	50.00	24.60	22.78	19.00	30.00	
enroll	40,309	50.00	37.12	28.82	31.00	45.00	
Total medical expenses	80,618	100.00	235,759	237,807	177,318	224,600	
Hospice care							<0.001
not enroll	40,309	50.00	257,877	277,004	189,194	251,797	
enroll	40,309	50.00	213,642	188,139	167,256	199,987	
**Variables**	**24 Weeks before Death**						
	**N**	**%**	**Mean**	**SD**	**Median**	**IQR**	*p*-Value *
Total morphine dose	35,622	100.00	2403.63	8849.40	40.00	990.00	
Hospice care							<0.001
not enroll	17,811	50.00	758.47	3941.51	10.00	160.00	
enroll	17,811	50.00	4048.78	11,648.15	247.00	3220.00	
Total length of stay	35,622	100.00	41.76	41.03	30.00	46.00	
Hospice care							<0.001
not enroll	17,811	50.00	32.87	32.94	24.00	36.00	
enroll	17,811	50.00	50.66	46.09	38.00	57.00	
Total medical expenses	35,622	100.00	377,152	362,781	293,162	364,676	
Hospice care							0.021
not enroll	17,811	50.00	389,052	405,138	298,017	376,796	
enroll	17,811	50.00	365,251	314,337	289,413	353,749	

* Mann–Whitney test.

**Table 2:** Effects of hospice care enrollment before death on the total morphine dose, total length of hospital stay, and total medical expenses incurred by patients with terminal cancer who enrolled and those who did not enroll in hospice care at 1, 4, 8, 12, and 24 weeks before death (by multiple regression models) *.

Variables	1 Week before Death				4 Weeks before Death				8 Weeks before Death				12 Weeks before Death				24 Weeks before Death			
	Ratio	95% CI		*p*-Value	Ratio	95% CI		*p*-Value	Ratio	95% CI		*p*-Value	Ratio	95% CI		*p*-Value	Ratio	95% CI		*p*-Value
Total morphine dose																				
enroll vs. not enroll(ref)	19.29	18.74	19.84	<0.001	29.19	28.08	30.34	<0.001	28.80	27.31	30.37	<0.001	27.89	26.04	29.87	<0.001	23.49	21.20	26.03	<0.001
Total length of stay																				
enroll vs. not enroll(ref) enroll(ref)	1.93	1.90	1.97	<0.001	2.11	2.06	2.15	<0.001	1.88	1.83	1.94	<0.001	1.73	1.67	1.78	<0.001	1.57	1.50	1.65	<0.001
Total medical expense																				
enroll vs. not enroll(ref) enroll(ref)	0.79	0.78	0.81	<0.001	0.86	0.85	0.87	<0.001	0.87	0.85	0.88	<0.001	0.90	0.89	0.92	<0.001	0.73	0.70	0.76	<0.001

* All models have been controlled for the relevant variables, including gender, age, education levels, marital status, monthly salary, urbanization of residence area, CCI, cancer type, primary medical organizations level, institutional ownership, and death year.

## Data Availability

Data are available from the Health and Welfare Data Science Center of the Ministry of Health and Welfare (MOHW) (https://www.mohw.gov.tw/mp-2.html) (accessed on 1 May 2023), Taiwan. All interested researchers can apply to use the database managed by the MOHW. Due to legal restrictions imposed by the Taiwanese government related to the Personal Information Protection Act, the database cannot be made publicly available. Raw data from the Health and Welfare Data Science Center cannot be brought out. The restrictions prohibited the authors from making the minimal data set publicly available.

## Supplementary Materials

The following supporting information can be downloaded at: https://www.mdpi.com/article/10.3390/healthcare11212867/s1, Table S1. Code for hospice-related payment; Table S2. Morphine medical order; Table S3. Proportion of terminal cancer patients receiving hospice care before death; Table S4. Comparison of the differences in patients with terminal cancer who enrolled and those who did not enroll in hospice care before matching; Table S5. Comparison of the differences in patients with terminal cancer who enrolled and those who did not enroll in hospice care at 1, 4, 8, 12, and 24 weeks before death incurred by matching; Table S6. Compare the average total morphine dose between terminal cancer patients enrolled in hospice care and those who did not, at 1, 4, 8, 12, and 24 weeks before death in each year; Table S7. Compare the average total length of stay between terminal cancer patients enrolled in hospice care and those who were not, at 1, 4, 8, 12, and 24 weeks before death in each year; Table S8. Compare the average total medical expenses of terminal cancer patients enrolled in hospice care versus those who do not, at 1, 4, 8, 12, and 24 weeks before death each year.
