# Supplementary material for: Effects of the Time of Hospice and Palliative Care Enrollment before Death on Morphine, Length of Stay, and Healthcare Expense in Patients with Cancer in Taiwan
Source: Healthcare (Basel). 2023 Oct 31;11(21):2867. doi: 10.3390/healthcare11212867 (PMC10648820; doi:10.3390/healthcare11212867)
Supplement: Supplementary file 1 [file healthcare-11-02867-s001.zip › healthcare-2677813-supplementary.pdf]

Supplementary Table S1. Code for hospice-related payment

Iterm	Codes
Hospice wards care management fee (every day)	05601K、05602A、05603B、03001KB、03002AB、03003BB、03004BB、05604K、05605A、05606B、P1101K、P1102A、P1103B、P1104K、P1105A、P1106B
Home-based hospice care / Community hospice care	05312C、05362C、05323C、05363C、05336C、05364C、05337C、05365C、05313C、05366C、05324C、05367C、05338C、05368C、05339C、05369C、05314C、05370C、05325C、05371C、05340C、05326C、05327C、05315C、05374C、05316C、05372C、05373C、05341C
Hospice shared care	P4401B、P4402B、P4403B
Palliative care family consultation	02020B

Supplementary Table S2. Morphine medical order

Iterm	Codes
Medical order of morphine	A005886209、A005891209、B021452243、B019000100、A042534100、B019001100、A005860100、A005869155、A005869199、A049758157、A049758199、A056306100、AC05860100、AC05886209、AC05891209、AC42534100、AC49758151、AC49758157、AC56306100、B021452299、A005852209、A005862209、BC19000100、BC19001100、BC26922209、BC27052212、A006235177、A006235199、B023000216、B023779100、BC23000216、BC23779100

Supplementary Table S3. Proportion of terminal cancer patients receiving hospice care before death

			Not enroll		Enroll	
Year	N	%	n_0	%	n_1	%
2005	33876	5.60	27848	82.21	6028	17.79
2006	34878	5.76	28069	80.48	6809	19.52
2007	38043	6.29	30540	80.28	7503	19.72
2008	39740	6.57	31859	80.17	7881	19.83
2009	40857	6.75	32237	78.90	8620	21.10
2010	42112	6.96	32623	77.47	9489	22.53
2011	43459	7.18	30839	70.96	12620	29.04
2012	44855	7.41	27607	61.55	17248	38.45
2013	45947	7.59	25528	55.56	20419	44.44
2014	47184	7.80	23777	50.39	23407	49.61
2015	48100	7.95	20405	42.42	27695	57.58
2016	48473	8.01	18426	38.01	30047	61.99
2017	49243	8.14	17528	35.59	31715	64.41
2018	48359	7.99	18131	37.49	30228	62.51
Total	605126	100.00	365417	60.39	239709	39.61

Supplementary Table S4. Comparison of the differences in patients with terminal cancer who enrolled and those who did not enroll in hospice care before matching

			Non-H		H group		$\chi2$
Variables	N	%	n_0	%	n_1	%	p-value
Total	605126	100.00	365417	60.39	239709	39.61	
**Gender**							<0.001
Male	380319	62.85	238437	62.69	141882	37.31	
Female	224807	37.15	126980	56.48	97827	43.52	
**Age group**							<0.001
<55 years	111438	18.42	65626	58.89	45812	41.11	
55-64 years	122092	20.18	69102	56.60	52990	43.40	
65-74 years	138623	22.91	83964	60.57	54659	39.43	
75-84 years	157967	26.10	99718	63.13	58249	36.87	
≧85 years	75006	12.40	47007	62.67	27999	37.33	
**Education Level**							<0.001
Elementary or below	328769	54.33	209145	63.61	119624	36.39	
Junior	113541	18.76	65461	57.65	48080	42.35	
Senior(Vocational)	124607	20.59	69630	55.88	54977	44.12	
College or above	38209	6.31	21181	55.43	17028	44.57	
**Marital status**							<0.001
Never married	43235	7.14	25138	58.14	18097	41.86	
Married	385515	63.71	234782	60.90	150733	39.10	
Divorced and Widowed	176376	29.15	105497	59.81	70879	40.19	
**Monthly salary (NTD)**							<0.001
≦17,280 (ref)	187919	31.05	112806	60.03	75113	39.97	
17,281-22,800	232626	38.44	147782	63.53	84844	36.47	
22,801-28,800	49294	8.15	26471	53.70	22823	46.30	
28,801-36,300	39310	6.50	22832	58.08	16478	41.92	
36,301-45,800	45272	7.48	26004	57.44	19268	42.56	
45,801-57,800	18389	3.04	10713	58.26	7676	41.74	
≧57,801	32316	5.34	18809	58.20	13507	41.80	
**Urbanization of residence area**							<0.001
1 (ref)	150306	24.84	88650	58.98	61656	41.02	
2	175104	28.94	102560	58.57	72544	41.43	
3	99203	16.39	59889	60.37	39314	39.63	
4	98648	16.30	62438	63.29	36210	36.71	
5	19430	3.21	12446	64.06	6984	35.94	
6	32750	5.41	20230	61.77	12520	38.23	
7	29685	4.91	19204	64.69	10481	35.31	
**CCI**							<0.001
0	158925	26.26	91574	57.62	67351	42.38	
1	162152	26.80	96607	59.58	65545	40.42	
2	108298	17.90	65763	60.72	42535	39.28	
3	80068	13.23	50007	62.46	30061	37.54	
>3	95683	15.81	61466	64.24	34217	35.76	

Supplementary Table S4. Comparison of the differences in patients with terminal cancer who enrolled and those who did not enroll in hospice care before matching (cont.)

			Non-H		H group		$\chi 2$
Variables	N	%	n_0	%	n_1	%	p-value
**Cancer type**							<0.001
Lung cancer	100230	16.56	61635	61.49	38595	38.51	
Liver cancer	100370	16.59	64262	64.03	36108	35.97	
Colorectal cancer	66508	10.99	39817	59.87	26691	40.13	
Breast cancer	25048	4.14	13545	54.08	11503	45.92	
Oral cancer	33823	5.59	19589	57.92	14234	42.08	
Prostate cancer	15536	2.57	10197	65.63	5339	34.37	
Stomach cancer	29437	4.86	17932	60.92	11505	39.08	
Pancreatic cancer	18772	3.10	9404	50.10	9368	49.90	
Esophagus cancer	18600	3.07	11708	62.95	6892	37.05	
Cervical cancer	8482	1.40	5048	59.51	3434	40.49	
Others	77027	12.73	48507	62.97	28520	37.03	
Multiple cancers	111293	18.39	63773	57.30	47520	42.70	
**multidisciplinary cancer care**							<0.001
No	468730	77.46	301103	64.24	167627	35.76	
Yes	136396	22.54	64314	47.15	72082	52.85	
**Institutional ownership level**							<0.001
Public	192654	31.84	110277	57.24	82377	42.76	
Nonpublic	412472	68.16	255140	61.86	157332	38.14	
**Primary healthcare organizations**							<0.001
Medical center	296872	49.06	174174	58.67	122698	41.33	
Regional hospital	232295	38.39	136389	58.71	95906	41.29	
District hospital	41438	6.85	30084	72.60	11354	27.40	
Clinic	34521	5.70	24770	71.75	9751	28.25	

Supplementary Table S5. Comparison of the differences in patients with terminal cancer who enrolled and those who did not enroll in hospice care at 1, 4, 8, 12, and 24 weeks before death incurred by matching

	1 week before death													
	Before matching							After 1：1 matching						
			Non-H		H group		χ^2			Non-H		H group		χ^2
Variables	N	%	n_0	%	n_1	%	p-value	N	%	n_0	%	n_1	%	p-value
Total	570082	100.00	365417	64.10	204665	35.90		336624	100.00	168312	50.00	168312	50.00	
**Gender**							<0.001							0.794
Male	358764	62.93	238437	65.25	120327	58.79		206269	61.28	103097	61.25	103172	61.30	
Female	211318	37.07	126980	34.75	84338	41.21		130355	38.72	65215	38.75	65140	38.70	
**Age groups**							<0.001							0.924
<55 years	105323	18.48	65626	17.96	39697	19.40		59423	17.65	29649	17.62	29774	17.69	
55-64 years	114856	20.15	69102	18.91	45754	22.36		69173	20.55	34562	20.53	34611	20.56	
65-74 years	130358	22.87	83964	22.98	46394	22.67		75897	22.55	37952	22.55	37945	22.54	
75-84 years	148748	26.09	99718	27.29	49030	23.96		87965	26.13	43984	26.13	43981	26.13	
≧85 years	70797	12.42	47007	12.86	23790	11.62		44166	13.12	22165	13.17	22001	13.07	
**CCI**							<0.001							0.995
0	149599	26.24	91574	25.06	58025	28.35		90702	26.94	45334	26.93	45368	26.95	
1	152672	26.78	96607	26.44	56065	27.39		90774	26.97	45360	26.95	45414	26.98	
2	102306	17.95	65763	18.00	36543	17.86		61126	18.16	30610	18.19	30516	18.13	
3	75183	13.19	50007	13.68	25176	12.30		43286	12.86	21633	12.85	21653	12.86	
>3	90322	15.84	61466	16.82	28856	14.10		50736	15.07	25375	15.08	25361	15.07	
**Cancer type**							<0.001							1.000
Lung cancer	94526	16.58	61635	16.87	32891	16.07		54991	16.34	27484	16.33	27507	16.34	
Liver cancer	93234	16.35	64262	17.59	28972	14.16		50546	15.02	25248	15.00	25298	15.03	
Colorectal cancer	62721	11.00	39817	10.90	22904	11.19		38016	11.29	19070	11.33	18946	11.26	
Breast cancer	23271	4.08	13545	3.71	9726	4.75		14352	4.26	7184	4.27	7168	4.26	
Oral cancer	32256	5.66	19589	5.36	12667	6.19		20251	6.02	10132	6.02	10119	6.01	
Prostate cancer	14774	2.59	10197	2.79	4577	2.24		8678	2.58	4360	2.59	4318	2.57	
Stomach cancer	27798	4.88	17932	4.91	9866	4.82		15911	4.73	7910	4.70	8001	4.75	
Pancreatic cancer	17369	3.05	9404	2.57	7965	3.89		10489	3.12	5252	3.12	5237	3.11	
Esophagus cancer	17700	3.10	11708	3.20	5992	2.93		9811	2.91	4914	2.92	4897	2.91	
Cervical cancer	8078	1.42	5048	1.38	3030	1.48		4581	1.36	2300	1.37	2281	1.36	
Others	73248	12.85	48507	13.27	24741	12.09		41663	12.38	20811	12.36	20852	12.39	
Multiple cancers	105107	18.44	63773	17.45	41334	20.20		67335	20.00	33647	19.99	33688	20.02	

	4 weeks before death													
	Before matching							After 1：1 matching						
			Non-H		H group		χ^2			Non-H		H group		χ^2
Variables	N	%	n_0	%	n_1	%	p-value	N	%	n_0	%	n_1	%	p-value
Total	480212	100.00	365417	76.09	114795	23.91		217080	100.00	108540	50.00	108540	50.00	
**Gender**							<0.001							0.724
Male	304056	63.32	238437	65.25	65619	57.16		127692	58.82	63805	58.78	63887	58.86	
Female	176156	36.68	126980	34.75	49176	42.84		89388	41.18	44735	41.22	44653	41.14	
**Age groups**							<0.001							0.952
<55 years	88959	18.52	65626	17.96	23333	20.33		41485	19.11	20690	19.06	20795	19.16	
55-64 years	94922	19.77	69102	18.91	25820	22.49		47371	21.82	23677	21.81	23694	21.83	
65-74 years	109290	22.76	83964	22.98	25326	22.06		48489	22.34	24243	22.34	24246	22.34	
75-84 years	126573	26.36	99718	27.29	26855	23.39		53016	24.42	26518	24.43	26498	24.41	

≧85 years	60468	12.59	47007	12.86	13461	11.73		26719	12.31	13412	12.36	13307	12.26	
**CCI**							<0.001							1.000
0	125062	26.04	91574	25.06	33488	29.17		61877	28.50	30917	28.48	30960	28.52	
1	128342	26.73	96607	26.44	31735	27.64		59616	27.46	29828	27.48	29788	27.44	
2	86443	18.00	65763	18.00	20680	18.01		39393	18.15	19696	18.15	19697	18.15	
3	63546	13.23	50007	13.68	13539	11.79		26158	12.05	13077	12.05	13081	12.05	
>3	76819	16.00	61466	16.82	15353	13.37		30036	13.84	15022	13.84	15014	13.83	
**Cancer type**							<0.001							1.000
Lung cancer	80258	16.71	61635	16.87	18623	16.22		35708	16.45	17832	16.43	17876	16.47	
Liver cancer	77214	16.08	64262	17.59	12952	11.28		25730	11.85	12867	11.85	12863	11.85	
Colorectal cancer	53093	11.06	39817	10.90	13276	11.56		24829	11.44	12413	11.44	12416	11.44	
Breast cancer	19235	4.01	13545	3.71	5690	4.96		10145	4.67	5085	4.68	5060	4.66	
Oral cancer	27783	5.79	19589	5.36	8194	7.14		15123	6.97	7565	6.97	7558	6.96	
Prostate cancer	13024	2.71	10197	2.79	2827	2.46		5528	2.55	2765	2.55	2763	2.55	
Stomach cancer	23287	4.85	17932	4.91	5355	4.66		9831	4.53	4903	4.52	4928	4.54	
Pancreatic cancer	13504	2.81	9404	2.57	4100	3.57		6941	3.20	3460	3.19	3481	3.21	
Esophagus cancer	15143	3.15	11708	3.20	3435	2.99		6521	3.00	3256	3.00	3265	3.01	
Cervical cancer	6922	1.44	5048	1.38	1874	1.63		3205	1.48	1612	1.49	1593	1.47	
Others	63103	13.14	48507	13.27	14596	12.71		27653	12.74	13831	12.74	13822	12.73	
Multiple cancers	87646	18.25	63773	17.45	23873	20.80		45866	21.13	22951	21.15	22915	21.11	

	8 weeks before death													
	Before matching							After 1：1 matching						
			Non-H		H group		χ^2			Non-H		H group		χ^2
Variables	N	%	n_0	%	n_1	%	p-value	N	%	n_0	%	n_1	%	p-value
Total	427710	100.00	365417	85.44	62293	14.56		123500	100.00	61750	50.00	61750	50.00	
**Gender**							<0.001							0.986
Male	273000	63.83	238437	65.25	34563	55.48		68962	55.84	34483	55.84	34479	55.84	
Female	154710	36.17	126980	34.75	27730	44.52		54538	44.16	27267	44.16	27271	44.16	
**Age groups**							<0.001							1.000
<55 years	78331	18.31	65626	17.96	12705	20.40		24810	20.09	12393	20.07	12417	20.11	
55-64 years	82960	19.40	69102	18.91	13858	22.25		27413	22.20	13717	22.21	13696	22.18	
65-74 years	97369	22.77	83964	22.98	13405	21.52		26696	21.62	13344	21.61	13352	21.62	
75-84 years	114232	26.71	99718	27.29	14514	23.30		29001	23.48	14505	23.49	14496	23.48	
≧85 years	54818	12.82	47007	12.86	7811	12.54		15580	12.62	7791	12.62	7789	12.61	
**CCI**							<0.001							1.000
0	109595	25.62	91574	25.06	18021	28.93		35805	28.99	17895	28.98	17910	29.00	
1	113649	26.57	96607	26.44	17042	27.36		33709	27.29	16854	27.29	16855	27.30	
2	77274	18.07	65763	18.00	11511	18.48		22750	18.42	11366	18.41	11384	18.44	
3	57320	13.40	50007	13.68	7313	11.74		14506	11.75	7262	11.76	7244	11.73	
>3	69872	16.34	61466	16.82	8406	13.49		16730	13.55	8373	13.56	8357	13.53	
**Cancer type**							<0.001							1.000
Lung cancer	71860	16.80	61635	16.87	10225	16.41		20353	16.48	10176	16.48	10177	16.48	
Liver cancer	70240	16.42	64262	17.59	5978	9.60		11949	9.68	5976	9.68	5973	9.67	
Colorectal cancer	47225	11.04	39817	10.90	7408	11.89		14630	11.85	7307	11.83	7323	11.86	
Breast cancer	17046	3.99	13545	3.71	3501	5.62		6917	5.60	3457	5.60	3460	5.60	
Oral cancer	24270	5.67	19589	5.36	4681	7.51		9276	7.51	4642	7.52	4634	7.50	
Prostate cancer	11997	2.80	10197	2.79	1800	2.89		3571	2.89	1785	2.89	1786	2.89	

	Stomach cancer	20574	4.81	17932	4.91	2642	4.24		5139	4.16	2570	4.16	2569	4.16	
	Pancreatic cancer	11216	2.62	9404	2.57	1812	2.91		3499	2.83	1752	2.84	1747	2.83	
	Esophagus cancer	13442	3.14	11708	3.20	1734	2.78		3396	2.75	1692	2.74	1704	2.76	
	Cervical cancer	6146	1.44	5048	1.38	1098	1.76		2066	1.67	1025	1.66	1041	1.69	
	Others	56704	13.26	48507	13.27	8197	13.16		16268	13.17	8140	13.18	8128	13.16	
	Multiple cancers	76990	18.00	63773	17.45	13217	21.22		26436	21.41	13228	21.42	13208	21.39	
		12 weeks before death													
		Before matching							After 1：1 matching						
				Non-H		H group		χ^2			Non-H		H group		χ^2
Variables		N	%	n_0	%	n_1	%	p-value	N	%	n_0	%	n_1	%	p-value
Total		405838	100.00	365417	90.04	40421	9.96		80618	100.00	40309	50.00	40309	50.00	
**Gender**								<0.001							0.927
	Male	260481	64.18	238437	65.25	22044	54.54		44064	54.66	22039	54.68	22025	54.64	
	Female	145357	35.82	126980	34.75	18377	45.46		36554	45.34	18270	45.32	18284	45.36	
**Age groups**								<0.001							1.000
	<55 years	73713	18.16	65626	17.96	8087	20.01		16033	19.89	8011	19.87	8022	19.90	
	55-64 years	77882	19.19	69102	18.91	8780	21.72		17529	21.74	8764	21.74	8765	21.74	
	65-74 years	92551	22.80	83964	22.98	8587	21.24		17168	21.30	8589	21.31	8579	21.28	
	75-84 years	109250	26.92	99718	27.29	9532	23.58		19056	23.64	9530	23.64	9526	23.63	
	≧85 years	52442	12.92	47007	12.86	5435	13.45		10832	13.44	5415	13.43	5417	13.44	
**CCI**								<0.001							1.000
	0	103065	25.40	91574	25.06	11491	28.43		22963	28.48	11479	28.48	11484	28.49	
	1	107555	26.50	96607	26.44	10948	27.08		21813	27.06	10905	27.05	10908	27.06	
	2	73381	18.08	65763	18.00	7618	18.85		15172	18.82	7583	18.81	7589	18.83	
	3	54766	13.49	50007	13.68	4759	11.77		9474	11.75	4734	11.74	4740	11.76	
	>3	67071	16.53	61466	16.82	5605	13.87		11196	13.89	5608	13.91	5588	13.86	
**Cancer type**								<0.001							1.000
	Lung cancer	68445	16.87	61635	16.87	6810	16.85		13610	16.88	6806	16.88	6804	16.88	
	Liver cancer	67879	16.73	64262	17.59	3617	8.95		7232	8.97	3616	8.97	3616	8.97	
	Colorectal cancer	44675	11.01	39817	10.90	4858	12.02		9700	12.03	4855	12.04	4845	12.02	
	Breast cancer	16097	3.97	13545	3.71	2552	6.31		5096	6.32	2549	6.32	2547	6.32	
	Oral cancer	22515	5.55	19589	5.36	2926	7.24		5816	7.21	2911	7.22	2905	7.21	
	Prostate cancer	11516	2.84	10197	2.79	1319	3.26		2625	3.26	1313	3.26	1312	3.25	
	Stomach cancer	19537	4.81	17932	4.91	1605	3.97		3176	3.94	1584	3.93	1592	3.95	
	Pancreatic cancer	10405	2.56	9404	2.57	1001	2.48		1964	2.44	981	2.43	983	2.44	
	Esophagus cancer	12729	3.14	11708	3.20	1021	2.53		1995	2.47	996	2.47	999	2.48	
	Cervical cancer	5750	1.42	5048	1.38	702	1.74		1380	1.71	682	1.69	698	1.73	
	Others	53855	13.27	48507	13.27	5348	13.23		10695	13.27	5347	13.27	5348	13.27	
	Multiple cancers	72435	17.85	63773	17.45	8662	21.43		17329	21.50	8669	21.51	8660	21.48	
		24 weeks before death													
		Before matching							After 1：1 matching						
				Non-H		H group		χ^2			Non-H		H group		χ^2
Variables		N	%	n_0	%	n_1	%	p-value	N	%	n_0	%	n_1	%	p-value
Total		383244	100.00	365417	95.35	17827	4.65		35622	100.00	17811	50.00	17811	50.00	
**Gender**								<0.001							0.907
	Male	247811	64.66	238437	65.25	9374	52.58		18758	52.66	9385	52.69	9373	52.62	
	Female	135433	35.34	126980	34.75	8453	47.42		16864	47.34	8426	47.31	8438	47.38	

**Age groups**							<0.001							1.000
<55 years	68907	17.98	65626	17.96	3281	18.40		6543	18.37	3268	18.35	3275	18.39	
55-64 years	72949	19.03	69102	18.91	3847	21.58		7683	21.57	3838	21.55	3845	21.59	
65-74 years	87731	22.89	83964	22.98	3767	21.13		7535	21.15	3768	21.16	3767	21.15	
75-84 years	103983	27.13	99718	27.29	4265	23.92		8532	23.95	4268	23.96	4264	23.94	
≧85 years	49674	12.96	47007	12.86	2667	14.96		5329	14.96	2669	14.99	2660	14.93	
**CCI**							<0.001							1.000
0	96413	25.16	91574	25.06	4839	27.14		9678	27.17	4839	27.17	4839	27.17	
1	101352	26.45	96607	26.44	4745	26.62		9488	26.64	4747	26.65	4741	26.62	
2	69194	18.05	65763	18.00	3431	19.25		6854	19.24	3427	19.24	3427	19.24	
3	52183	13.62	50007	13.68	2176	12.21		4337	12.18	2167	12.17	2170	12.18	
>3	64102	16.73	61466	16.82	2636	14.79		5265	14.78	2631	14.77	2634	14.79	
**Cancer type**							<0.001							1.000
Lung cancer	64691	16.88	61635	16.87	3056	17.14		6109	17.15	3053	17.14	3056	17.16	
Liver cancer	65713	17.15	64262	17.59	1451	8.14		2902	8.15	1451	8.15	1451	8.15	
Colorectal cancer	41978	10.95	39817	10.90	2161	12.12		4326	12.14	2167	12.17	2159	12.12	
Breast cancer	14946	3.90	13545	3.71	1401	7.86		2805	7.87	1404	7.88	1401	7.87	
Oral cancer	20720	5.41	19589	5.36	1131	6.34		2253	6.32	1126	6.32	1127	6.33	
Prostate cancer	10901	2.84	10197	2.79	704	3.95		1411	3.96	707	3.97	704	3.95	
Stomach cancer	18537	4.84	17932	4.91	605	3.39		1209	3.39	604	3.39	605	3.40	
Pancreatic cancer	9718	2.54	9404	2.57	314	1.76		622	1.75	308	1.73	314	1.76	
Esophagus cancer	12091	3.15	11708	3.20	383	2.15		750	2.11	373	2.09	377	2.12	
Cervical cancer	5374	1.40	5048	1.38	326	1.83		642	1.80	320	1.80	322	1.81	
Others	50876	13.28	48507	13.27	2369	13.29		4740	13.31	2371	13.31	2369	13.30	
Multiple cancers	67699	17.66	63773	17.45	3926	22.02		7853	22.05	3927	22.05	3926	22.04	

H group: Advanced cancer patients who enrolled in palliative and hospice care (H group) and who did not (non-H group).

Supplementary Table S6. Compare the average total morphine dose between terminal cancer patients enrolled in hospice care and those who did not, at 1, 4, 8, 12, and 24 weeks before death in each year

	1 week before death			4 weeks before death			8 weeks before death			12 weeks before [illegible]			24 weeks before death		
	Non-H	H		Non-H	H group		Non-H	H group		Non-H	H group		Non-H	H group	
	mean[a]	mean[b]	b/a	mean[a]	mean[b]	b/a	mean[a]	mean[b]	b/a	mean[a]	mean[b]	b/a	mean[a]	mean[b]	b/a
Total morphine dose															
2005	77.82	243.93	3.13	279.85	1214.72	4.34	503.04	2465.78	4.90	561.75	3431.60	6.11	513.42	5727.91	11.16
2006	72.19	252.37	3.50	297.82	1266.82	4.25	524.88	2861.57	5.45	665.05	4445.34	6.68	1017.66	7087.38	6.96
2007	80.63	263.61	3.27	334.10	1239.98	3.71	640.87	2703.16	4.22	836.58	4186.85	5.00	1019.71	7636.17	7.49
2008	76.30	252.70	3.31	322.09	1228.71	3.81	547.80	2560.98	4.68	676.92	3771.30	5.57	942.96	6207.65	6.58
2009	84.01	237.34	2.83	350.38	1182.21	3.37	641.04	2409.78	3.76	866.41	3821.68	4.41	1084.52	7574.86	6.98
2010	76.22	231.86	3.04	286.35	1117.92	3.90	506.25	2431.32	4.80	638.66	3651.53	5.72	774.27	7800.37	10.07
2011	79.60	208.97	2.63	318.16	991.41	3.12	543.49	2166.99	3.99	610.08	3420.49	5.61	707.84	6918.33	9.77
2012	70.10	193.74	2.76	281.03	914.61	3.25	482.69	1937.51	4.01	606.71	2931.81	4.83	811.65	4870.85	6.00
2013	68.02	177.21	2.61	285.41	869.57	3.05	533.25	1892.41	3.55	702.44	2758.05	3.93	1008.42	4781.59	4.74
2014	60.90	157.41	2.58	266.01	762.83	2.87	459.05	1621.98	3.53	597.88	2481.25	4.15	794.20	4128.77	5.20
2015	57.53	144.18	2.51	242.84	709.71	2.92	445.39	1536.69	3.45	592.22	2265.80	3.83	740.75	3749.07	5.06
2016	58.59	136.99	2.34	237.49	674.03	2.84	430.54	1483.83	3.45	547.25	2126.96	3.89	715.29	3684.08	5.15
2017	52.11	130.47	2.50	205.24	608.97	2.97	385.94	1294.91	3.36	496.36	1858.18	3.74	736.13	3063.44	4.16
2018	54.75	128.20	2.34	207.17	592.76	2.86	378.70	1208.33	3.19	490.06	1686.03	3.44	626.86	2644.47	4.22

H group: Advanced cancer patients who enrolled in palliative and hospice care (H group) and who did not (non-H group).

Supplementary Table S7. Compare the average total length of stay between terminal cancer patients enrolled in hospice care and those who were not, at 1, 4, 8, 12, and 24 weeks before death in each year

	1 week before death			4 weeks before death			8 weeks before death			12 weeks before death			24 weeks before death		
	Non-H	H group		Non-H	H group		Non-H	H group		Non-H	H group		Non-H	H group	
	mean[a]	mean[b]	b/a	mean[a]	mean[b]	b/a	mean[a]	mean[b]	b/a	mean[a]	mean[b]	b/a	mean[a]	mean[b]	b/a
**Length of stay**															
**Year at death**															
2005	4.90	5.00	1.02	15.97	17.24	1.08	23.94	27.01	1.13	30.22	34.22	1.13	41.30	49.05	1.19
2006	4.92	5.20	1.06	16.11	18.47	1.15	24.44	29.81	1.22	30.61	37.61	1.23	41.19	49.71	1.21
2007	5.01	5.18	1.03	16.43	18.38	1.12	25.93	29.07	1.12	31.96	36.09	1.13	41.73	51.88	1.24
2008	4.93	5.27	1.07	16.03	18.71	1.17	24.83	29.15	1.17	29.76	36.57	1.23	42.17	46.26	1.10
2009	5.05	5.22	1.03	16.52	18.55	1.12	25.17	29.88	1.19	30.69	36.45	1.19	41.92	49.36	1.18
2010	5.01	5.27	1.05	16.16	18.60	1.15	25.02	28.75	1.15	31.52	34.13	1.08	43.13	46.92	1.09
2011	4.85	5.50	1.13	15.56	19.55	1.26	23.43	30.79	1.31	29.40	36.01	1.22	39.26	46.53	1.19
2012	4.62	5.64	1.22	14.42	20.19	1.40	21.85	32.19	1.47	27.26	39.48	1.45	37.56	54.10	1.44
2013	4.49	5.65	1.26	14.09	20.14	1.43	21.54	31.48	1.46	26.11	39.37	1.51	35.29	53.26	1.51
2014	4.31	5.64	1.31	13.31	20.12	1.51	20.13	31.76	1.58	24.90	39.21	1.58	32.80	54.39	1.66
2015	4.11	5.54	1.35	12.50	19.53	1.56	18.61	30.88	1.66	22.75	38.50	1.69	30.62	52.29	1.71
2016	3.92	5.47	1.39	11.91	19.04	1.60	17.97	29.69	1.65	22.11	36.69	1.66	30.83	49.91	1.62
2017	3.81	5.42	1.42	11.82	18.73	1.58	17.73	29.13	1.64	21.47	36.29	1.69	29.01	50.89	1.75
2018	3.99	5.37	1.35	12.58	18.59	1.48	18.85	28.79	1.53	22.94	35.46	1.55	30.90	48.46	1.57

H group: Advanced cancer patients who enrolled in palliative and hospice care (H group) and who did not (non-H group).

Supplementary Table S8. Compare the average total medical expenses of terminal cancer patients enrolled in hospice care versus those who do not, at 1, 4, 8, 12, and 24 weeks before death each year

	1 week before death			4 weeks before death			8 weeks before death			12 weeks before death			24 weeks before death		
	Non-H	H group		Non-H	H group		Non-H	H group		Non-H	H group		Non-H	H group	
	mean[a]	mean[b]	b/a	mean[a]	mean[b]	b/a	mean[a]	mean[b]	b/a	mean[a]	mean[b]	b/a	mean[a]	mean[b]	b/a
**Total costs**															
**Year at death**															
2005	39925	17189	0.43	121086	62633	0.52	183421	112206	0.61	244228	158274	0.65	367907	275196	0.75
2006	41279	16375	0.40	122603	62109	0.51	194050	118907	0.61	248975	172476	0.69	384904	300006	0.78
2007	41329	16664	0.40	124265	61352	0.49	195154	113013	0.58	253897	161734	0.64	385898	291626	0.76
2008	41769	16798	0.40	123102	62971	0.51	196785	116868	0.59	250138	164641	0.66	383420	286780	0.75
2009	43558	16146	0.37	128988	60949	0.47	199217	116175	0.58	260283	170440	0.65	383510	313056	0.82
2010	41564	15114	0.36	125022	56504	0.45	196455	103971	0.53	261773	148673	0.57	399697	254801	0.64
2011	42425	20301	0.48	126299	73106	0.58	193772	123070	0.64	250460	160386	0.64	378128	264770	0.70
2012	42191	24091	0.57	122261	85721	0.70	191313	143146	0.75	250962	194020	0.77	369600	324983	0.88
2013	44086	27386	0.62	126872	95260	0.75	200363	150699	0.75	256828	205837	0.80	389180	341590	0.88
2014	43689	29119	0.67	124685	99436	0.80	195146	157807	0.81	256535	211344	0.82	379602	350863	0.92
2015	43605	30995	0.71	123191	102684	0.83	187901	163074	0.87	243604	223001	0.92	367498	357037	0.97
2016	45391	32655	0.72	130455	107771	0.83	202939	170203	0.84	258695	230129	0.89	382992	384307	1.00
2017	45656	33115	0.73	130607	109846	0.84	202391	171385	0.85	256330	233006	0.91	386139	403680	1.05
2018	46062	33659	0.73	137513	111238	0.81	217447	177653	0.82	278223	241664	0.87	422189	407083	0.96

H group: Advanced cancer patients who enrolled in palliative and hospice care (H group) and who did not (non-H group).
